# Coherently Radiating Periodic Structures to Reduce the Number of Phase Shifters in a 2-D Phased Array

**DOI:** 10.3390/s21196592

**Published:** 2021-10-02

**Authors:** Elizvan Juárez, Marco A. Panduro, David H. Covarrubias, Alberto Reyna

**Affiliations:** 1 CICESE Research Center, Electronics and Telecommunications Department, Carretera Ensenada-Tijuana No. 3918, Zona Playitas, Ensenada 22860, Mexico; elizvan@cicese.edu.mx (E.J.); dacoro@cicese.mx (D.H.C.); 2 UAMRR-R, Universidad Autónoma de Tamaulipas, Carretera Reynosa-San Fernando, Reynosa 88779, Tamaulipas, Mexico; alberto.reyna@docentes.uat.edu.mx

**Keywords:** 2-D phased array, coherently radiating periodic structures, phase shifter, side lobe level, beam-scanning

## Abstract

This paper illustrates the application of CORPS (coherently radiating periodic structures) for feeding 2-D phased arrays with a reduced number of phase shifter (PS) devices. Three design configurations using CORPS are proposed for 2-D phased arrays. The design model of phased array for these configurations considers the cophasal excitation required for this structure to set a strategic way for feeding the antenna elements and reducing the number of PS devices. Blocks of 2 × 3 and 4 × 7 CORPS networks depending on the configuration in the 2-D phased array are set strategically in the feeding network to generate the cophasal excitation required in the antenna elements. These design configurations used for feeding the antenna elements in the planar array geometry provide several advantages with respect to others in the scanning capability and the reduction of the number of PS devices of the array system. The full-wave simulation results for the proposed configurations in 2-D phased arrays provide a reduction in the number of PSs of until 69% for a scanning range of ±25° in elevation and ±40° in azimuth. The application of the raised cosine amplitude distribution could generate radiation patterns with a SLL_PEAK ≈ −19 dB and SLL_PEAK ≈ −23 dB for the design proposed configurations in all the scanning range.

## 1. Introduction

Antenna arrays are a very important element of wireless communication systems. Many of new generation systems will be based on different antenna arrays structures [1]. Therefore, it is important to generate new design techniques for reducing the complexity and the cost of the antenna array system maintaining an optimal radiation performance.

The state of art includes two recent techniques for simplifying the number of phase shifters (PS): the subarrays technology [1,2,3,4,5,6,7,8] and the CORPS technology (coherently radiating periodic structures) [9,10]. The subarrays technology considers randomly grouped subarrays in linear and planar arrays for a reduced number of PS. This technique reduces the number of PS devices for limited scan-angle phased arrays [1,11]. Furthermore, the CORPS technique has been applied successfully in several previous studies for simplifying the beamforming network in different antenna array geometries [9,10,12,13]. Although these previous works deal with the application of CORPS for different antenna array geometries, the evaluation and proposal of new design techniques for reducing the PS devices are still scarce. The design technique presented in [10] illustrated the reduction of PS devices for linear phased arrays using CORPS. However, more study and analysis is required to generate new and more design configurations with different antenna array geometries to simplify the beamforming network by reducing the number of PS with a wide range of beam-scanning and low SLL. These new design techniques and configurations of phased array systems could be useful for the new generation of communication systems.

This paper presents the application of CORPS for feeding 2-D phased arrays with a reduced number of PS devices. This paper is an extension to the 2D case of the previous results published in [10]. However, this extension to consider 2-D phased arrays is not easy. This paper proposes new design configurations for 2-D phased arrays. Each block of the beamforming network is interconnected to generate the required phase plane by the 2D array with less PS devices. Each design configuration can generate the phase plane for beam-scanning.

The contributions of this paper and the differences with respect to previous work are as follows. Three design configurations using CORPS are proposed for 2-D phased arrays. The design model of phased array for these configurations considers the cophasal excitation required for this structure to set a strategic way for feeding the antenna elements and reducing the number of PS devices. The three proposed configurations for 2-D array structures provide a better scanning capability with respect to other existing configurations. These proposed design configurations utilize blocks of 2 × 3 and 4 × 7 CORPS networks depending on the configuration in the 2-D phased array system. These blocks of CORPS networks are set strategically in the feeding network to generate the cophasal excitation required in the antenna elements. These design configurations used for feeding the antenna elements in the planar array geometry provide several advantages with respect to others in the scanning capability and the reduction of the number of phase shifters of the array system. The full-wave simulation results for the proposed configurations in 2-D phased arrays provide a reduction (in the number of PSs) of until 69% for a scanning range of ±25° in elevation and ±40° in azimuth. The application of the raised cosine amplitude distribution could generate radiation patterns with a $SLL_{PEAK}\;\approx -19$ dB and $SLL_{PEAK}\;\approx -23$ dB for the design proposed configurations in all the scanning range.

## 2. Phased Antenna Array Model

We study the impact of the CORPS technology to reduce the number of phase shifter devices in 2-D phased arrays. Thus, it is important to analyze the geometry requirements to set the design configurations including the feeding network.

### 2.1. Design Configurations for 2-D Phased Arrays

**Figure 1:**
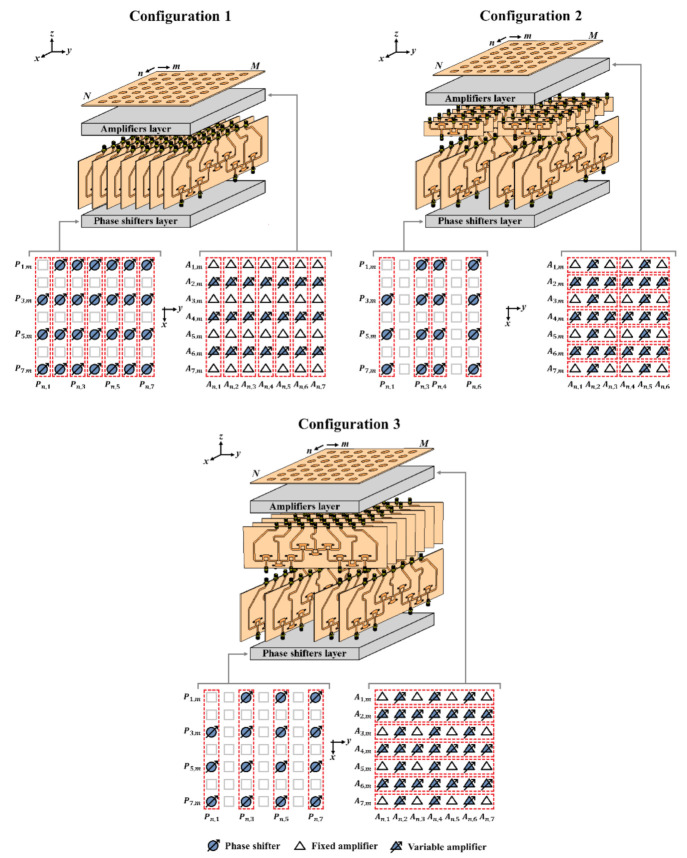
Planar array configurations using blocks of 2 × 3 and 4 × 7 CORPS networks.

We propose three interesting configurations using CORPS technology for planar arrays (illustrated in Figure 1). The array factor of a planar array with uniform separation is calculated as a function of θ and ϕ by using the next expression [14]:

(1) $$AF\left(\theta ,\phi \right)=\overset{N}{\underset{n=1}{\sum }}\overset{M}{\underset{m=1}{\sum }}I_{n,m}e^{j\left[kd\left(n-1\right)\psi_{x}+kd\left(m-1\right)\psi_{y}+\beta_{x}+\beta_{y}\right]}$$

where

(2) $$\psi_{x}=sin\left(\theta \right)\cos\left(\phi \right)$$

(3) $$\psi_{y}=sin\left(\theta \right)\sin\left(\phi \right)$$

(4) $$\beta_{x}=-kd\left(n-1\right)\sin\left(\theta_{0}\right)\cos\left(\phi_{0}\right)$$

(5) $$\beta_{y}=-kd\left(m-1\right)\sin\left(\theta_{0}\right)\sin\left(\phi_{0}\right)$$

*N* and *M* are the number of elements over the *x* and *y* axis, respectively, *I_nm_* is the amplitude excitation of the *nm*-th element of the array, and $\beta_{x}$ and $\beta_{y}$ are the cophasal excitation values required for beam-scanning to the desired directions of ($\theta_{0}$, $\phi_{0}$).

The configuration 1 proposes 7 blocks of 4 × 7 CORPS networks parallel to the *x-z* plane. This configuration uses 27 PSs and 21 variable amplifiers to control 49 antenna elements (7 × 7 array). This configuration could provide a reduction of 45% in the total number of PSs (in the elevation plane *x-z* plane) with a scanning range of (±25°) in the elevation plane and (±40°) in the azimuth plane (*y-z* plane).

Configuration 2 proposes the interconnection of the outputs of seven blocks of 4 × 7 CORPS networks (parallel to the *x-z* plane) to the inputs of blocks of 2 × 3 CORPS network. This configuration uses 15 PSs and 26 variable amplifiers to control 42 antenna elements (7 × 6 array). This configuration could provide a PSs reduction of 64% with a scanning range of (±25°) in the elevation plane and (±25°) in the azimuth plane.

Configuration 3 uses four blocks of 4 × 7 CORPS networks parallel to the *x-z* plane and the seven outputs of each block are connected to the inputs of a second layer of blocks 4 × 7 CORPS networks parallel to *y-z* plane. This configuration controls 49 antenna elements by using 15 PSs and 33 variable amplifiers. This configuration could present a PSs reduction of 69% with a scanning range of (±25°) in elevation and (±25°) in azimuth.

**Figure 2:**
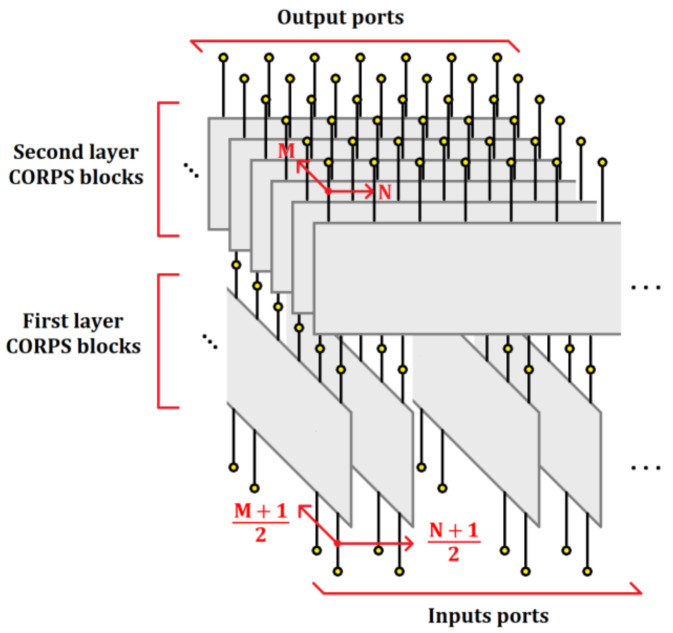
Block diagram of the beamforming network for the configuration 3 using 4 × 7 CORPS networks.

**Figure 3:**
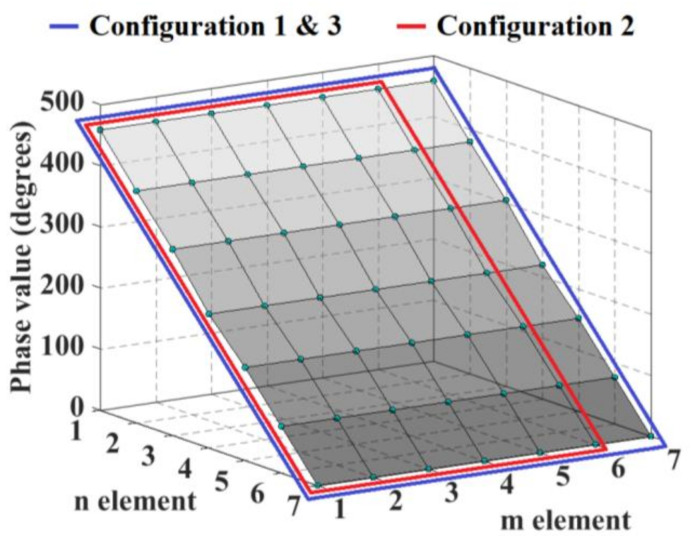
Phase plane generated for each design configuration of 2-D phased arrays.

The blocks of 2 × 3 and 4 × 7 CORPS networks are set strategically in each configuration to generate a cophasal excitation at the antenna elements. For example, Figure 2 illustrates the block diagram of the beamforming network for the configuration 3 (using 4 × 7 CORPS networks). Each block (in each layer) of the beamforming network is interconnected (in the way shown in Figure 1 and Figure 2) to generate the required phase plane by the 2D array, i.e., the same phase plane (that could be set considering the same number of PS devices to the number of antenna elements—the conventional case of progressive phase) is generated with less PS devices. Figure 3 shows the phase plane generated for each design configuration of 2-D phased arrays. Each design configuration can generate the phase plane for beam-scanning in the ranges previously mentioned.

The phase values of $P_{n,m}$ (at the input ports) for the three configurations are calculated by using the next expression:

(6) $$P_{n,m}=\beta_{x_{n}}+\beta_{y_{m}}$$

If the array center is considered as the phase reference (origin) and $d_{n,m}$ the distance from the origin to the antenna element, we can calculate the raised cosine distribution for the values of $I_{n,m}$ by using the next equation:

(7) $$I_{n,m}=\frac{1+\cos\left(\frac{d_{n,m}cos^{-1}\left(2a-1\right)}{0.5L}\right)}{2}$$

where *L* is the array longitude and

(8) $$d_{n,m}=\sqrt{dx_{n,m}^{2}+dy_{n,m}^{2}}$$

with $dx_{n,m}$ and $dy_{n,m}$ as the distance from the center of the array to the antenna element over the *x* and *y* axis, respectively.

The value of *a* for each configuration was calculated to obtain a ${\mathrm{SLL}}_{\mathrm{PEAK}}$ = −20 dB in all the scanning range (elevation and azimuth). It was selected a value of a = 0.19 for configurations 1 and 3 considering a 7 × 7 planar array. The normalized values of $I_{n,m}$ are illustrated in Table 1. Configuration 2 requires a smaller value of a = 0.14 due to the array is not symmetrical. This deteriorates the performance of the raised cosine distribution (in the SLL reduction) for the planar array with a fewer number of antenna elements. The normalized values of $I_{n,m}$ are illustrated in Table 2.

Therefore, the PSs are set at the input ports of the proposed configurations to generate the phase plane required for beam-scanning in elevation and azimuth, and the amplifiers set at the output ports of the network provide a raised cosine amplitude distribution that reduces the SLL. It is necessary to adjust the amplitude levels of the signals obtained at the outputs of the blocks of 4 × 7 CORPS networks to achieve an adequate raised cosine distribution for the array. First, we calculate the fixed amplification values (these values remain fixed during beam-scanning). Then, the variable amplification values are obtained for each beam-scanning direction.

### 2.2. Blocks of CORPS Feeding Networks

**Figure 4:**
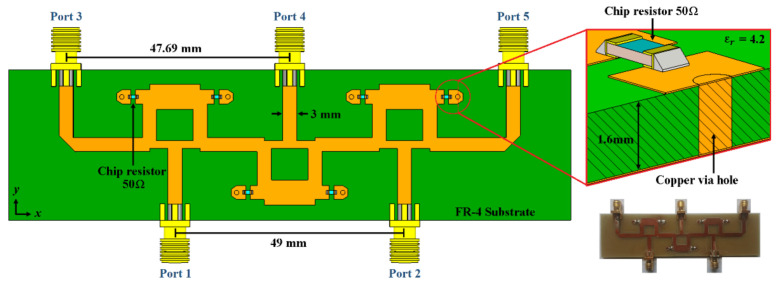
Block of 2 × 3 CORPS feeding network set to a design frequency of 6 GHz.

The proposed design configurations for 2-D phased arrays (explained in the previous section) use blocks of 2 × 3 and 4 × 7 CORPS networks to generate the phase plane required for beam-scanning in elevation and azimuth. Therefore, these blocks are designed, simulated, and fabricated for analyzing the proposed design configurations. Then, CST Microwave Studio is used to design and make simulations in a central frequency of 6 GHz. Figure 4 shows the block of 2 × 3 CORPS network. Three Gysel power dividers [15] with two resistances of 50 Ohms (FC0603) of surface mount technology are used in this configuration. Each power divider works as S or R nodes for the signals received from the two input ports. The substrate (FR4) presents dimensions of 120 mm × 32 mm and a thick of 1.6 mm with relative permittivity ε𝑟 = 4.2, 𝜇*_r_* = 1.0 and tangent loss of 0.025. More considerations about radiation efficiency can be achieved in FR4 such as in [16] or using others high performance substrates. However, this out of the scope of this work. Figure 4 shows the prototype of the 2 × 3 CORPS feeding network.

**Figure 5:**
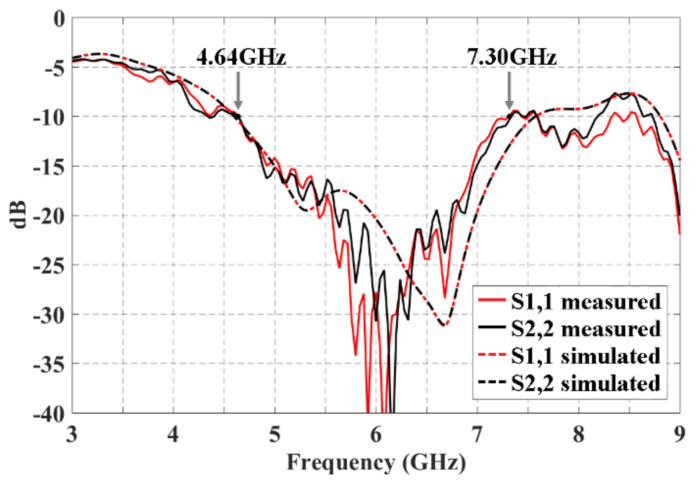
Reflection coefficients obtained by full-wave simulation and measured experimentally for the block of 2 × 3 CORPS network.

Figure 5 shows the reflection coefficients measured and simulated for the 2 × 3 CORPS network. The bandwidth measured is of 2.66 GHz (values below −10 dB) from 4.64 GHz to 7.30 GHz. It is obtained a value of S1,1 and S2,2 ≈ −28 dB in the design frequency (6 GHz). The lowest value of S1,1 and S2,2 is obtained at 6.12 GHz with a value of ≈−33 dB. The bandwidth obtained by simulation (2.68 GHz) and measurements are very similar with a slight displacement in high frequency of ≈250 MHz. The measured results agree with the electromagnetic simulation results with a certain divergence in high frequencies.

**Figure 6:**
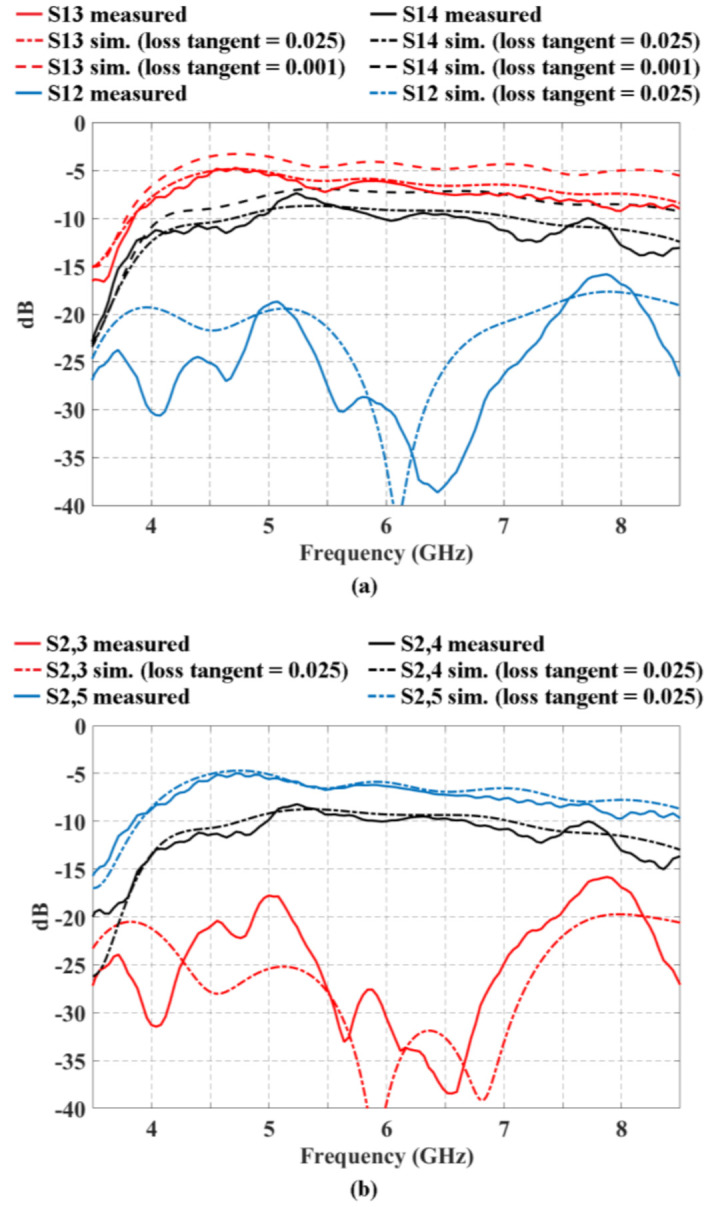
Behavior of the transmission coefficients for the block of 2 × 3 CORPS network using the substrate FR4 for a tangent loss of 0.025 and its comparison with respect to a value of tangent loss of 0.001.

Figure 6 illustrates the transmission coefficients (for the block of 2 × 3 CORPS network) simulated and measured in the ports 3, 4, and 5 (that represents the output ports 1, 2 and 3) when a signal is fed in the input port 1 (Figure 6a) and in the input port 2 (Figure 6b). These figures can illustrate a behavior of the insertion loss versus frequency. The parameters S_1,3_ and S_2,5_ present a value of ≈−6 dB in 6 GHz, i.e., a value of ≈−3 dB corresponds to the signal split in the S node, ≈−2 dB to the dissipation losses in the network and ≈−1 dB to the SMA connectors losses. It is obtained a value of S_1,4_ and S_2,4_ of ≈−10 dB, i.e., a value of ≈−3 dB corresponds to the signal split in the S node, ≈−3 dB to the recombination node, ≈−3 dB to the dissipation losses in the network and ≈−1 dB to the SMA connectors losses. It is obtained a very low value for the parameters S_1,5_ and S_2,3_ (≈−30 dB). This is because of the signal has not a direct way to the output ports analyzed. Furthermore, Figure 6 shows the behavior using the substrate FR4 for a tangent loss of 0.025 and its comparison with respect to a value of tangent loss of 0.001. As shown in this figure, there is a slight improvement (difference) of ≅2 dB for the case of the tangent loss of 0.001 at the frequency of 6 GHz. The measured results agree with the electromagnetic simulation results with a certain divergence in high frequencies.

**Figure 7:**
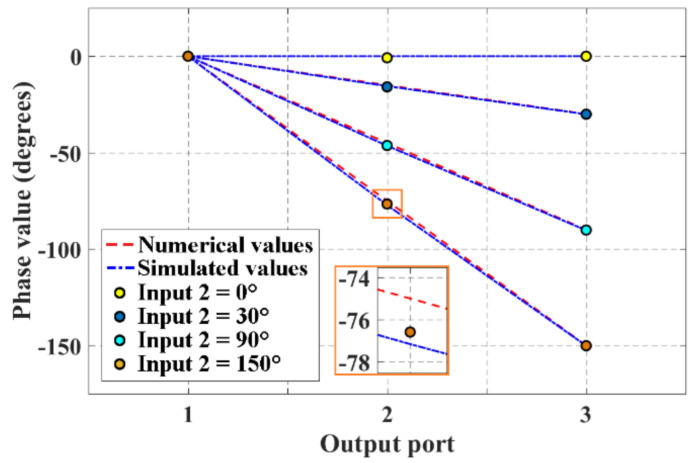
Phase values obtained by measurements and full-wave simulation for the block of 2 × 3 CORPS network.

Furthermore, two sinusoidal signals were full-wave simulated at the input ports of the network in order to determine the accuracy of the phase values generated by the block of 2 × 3 CORPS network. It was set a phase value of 0° at the input port 1 (as a fixed value), and the phase value at the input port 2 was varied. This generates a phase slope at the three output ports (i.e., an average phase value is delivered at the output port 2, a product of the recombination at the R node of the two signals received at its two input ports). Figure 7 shows the phase values obtained by the full-wave simulation and measurements. This figure illustrates a good agreement between the phase values obtained by simulation and measurements with an adequate recombination of the signal for different phase values at input port 2 (from 0° to 150°). The highest value of error is obtained for a phase value of 150° at the input port 2, i.e., an error of 1.2% (0.9°) is obtained with respect to the expected phase value (75°).

The block of 2 × 3 CORPS network operates in an expected way as illustrated in the behavior of the reflection and transmission coefficients and the phase values generated. Therefore, we can use this configuration of 2 × 3 CORPS network to analyze and study the block of 4 × 7 CORPS feeding network.

The block of 4 × 7 CORPS is designed to provide the phase interpolation property of a CORPS network of one layer as explained in detail in [10]. Table 3 illustrates (from results presented in [10]) a bandwidth from 4.76 GHz to 7.36 GHz (2.6 GHz). The behavior shows a maximum value of ≈−19.6 dB in the design frequency (6 GHz). As reported in [10], the ports 5 and 11 present values of ≈−8.2 dB (simulation) and ≈−8.5 dB (measurements). The port 8 presents ≈−15. The output ports that result from a recombination node (i.e., ports 6, 8 and 10) could present a different value depending on the phase difference between the two recombination signals. This makes necessary the use of variable amplifiers to generate an adequate distribution of amplitude at the outputs of the network [10].

**Figure 8:**
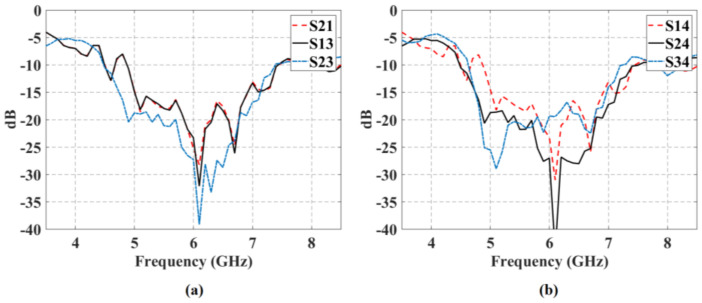
Isolation values versus frequency for the block of 4 × 7 CORPS.

The isolations versus frequency between each input and each output port are shown in Figure 8. The isolation values are greater than 19 dB (greater than 22 dB for the next ports: Input 1-Output 3, Input 2-Output 1, Input 2-Output 3) and the 10-dB bandwidth is greater than 2 GHz.

**Figure 9:**
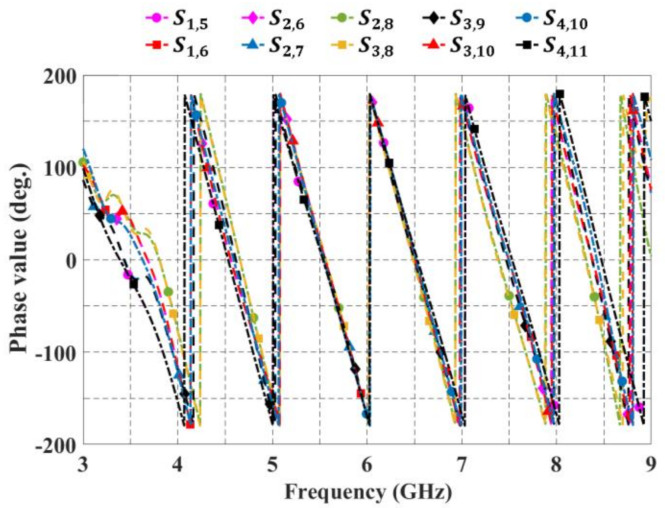
Phase response versus frequency for the block of 4 × 7 CORPS.

Figure 9 shows the phase response versus frequency of the block of 4 × 7 CORPS. All of these results match well with the design theory to illustrate that the blocks of 2 × 3 and 4 × 7 CORPS networks can be used in the 2-D phased array designs to achieve beamforming network functions.

## 3. Results and Discussion

The three proposed design configurations for 2-D phased arrays were analyzed to study the PS devices reduction performance and scanning capabilities. It is considered uniform separation of *d* = 0.5λ with scanning capabilities in both planes (elevation and azimuth). The full-wave simulations by CST electromagnetic solver shows that the required amplification values varies (slightly) during beam-scanning (≈5.2% for the configuration 1, ≈6.4% for the configuration 2 and ≈8.8% for the configuration 3).

The amplitude error (of the raised cosine distribution) is lower for configurations 2 and 3 since less fixed amplifiers are used. The variable amplifiers compensate the amplitude error in the network. In order to minimize the deterioration of the radiation pattern caused by the amplitude error at the outputs of the fixed amplifiers during beam-scanning, the fixed amplification values are calculated by averaging the amplification values of each amplifier for all scanning directions analyzed.

Table 4 shows the maximum values of the variable amplifiers and maximum values of phase error for each 2-D phased array configuration. The maximum value of variable amplification (obtained by full-wave simulation) for configuration 1 is of 7.98 for all scanning directions. The phase error for this configuration depends only on the performance of each independent 4 × 7 CORPS network. Therefore, the deterioration in the phase plane presented at the output ports is very low. The maximum value of the phase error obtained for this configuration is 4.1% (9.4°). The values of the variable amplifiers for configuration 1 increase as the main beam is scanned far from broadside (or the natural response), i.e., it is reached a maximum amplification value when the main beam is scanned in the direction of $\phi_{0}$ = 0° and $\theta_{0}$ = 25°.

In the case of configuration 2 the maximum value of variable amplification is of 8.89 and the maximum value of phase error is of 4.2% (9.6°) in all the scanning range. The average amplitude level obtained at the network output is 44% lower than the average amplitude level of configuration 1. This is due to the number of input ports is lower (a lower power level is introduced in the network) and it is used an additional layer of blocks of 2 × 3 CORPS networks increasing the signal attenuation. This is considered in the calculation of the raised cosine amplitude distribution to obtain the optimal amplification values.

The configuration 3 presents the highest signal attenuation. This is due to this configuration uses two layers of blocks of 4 × 7 CORPS networks. The maximum value of variable amplification for this configuration is of 11.28 (amplification level) and the maximum value of phase error is of 4.7% (10.7°).

Then, the proposed configurations were full-wave simulated in CST Microwave Studio in order to verify the performance considering mutual coupling and the amplitude and phase errors generated by each feeding network system. The full-wave simulations consider a circular patch as antenna element with a central frequency of 6 GHz and dimensions of: *r* = 13.02 mm, *h* = 1.6 mm (FR4 substrate) and $p^{\prime }$ = 2.07 [14]. The full wave simulation (by CST Microwave Studio) for each design configuration of 2-D phased arrays is achieved by blocks or layers. The simulation starts from the first layer of the input ports where the PSs values are set to generate the phase plane required for beam-scanning in elevation and azimuth. The phase error and amplitude error are estimated by the CST simulation (i.e., the error in each block of each configuration is calculated). Then these error values are taken to the next layers until the layer of amplifiers and the 2D antenna array. Furthermore, the SMA connectors and the resistances are considered in the full wave simulation. The raised cosine current distribution is generated at the antenna elements in a normalized way. The variable amplifiers (set at the recombination node outputs of the CORPS blocks) adjust the required amplitude values. The small unbalances generated by the fixed amplifiers are considered in the CST full-wave simulation.

**Figure 10:**
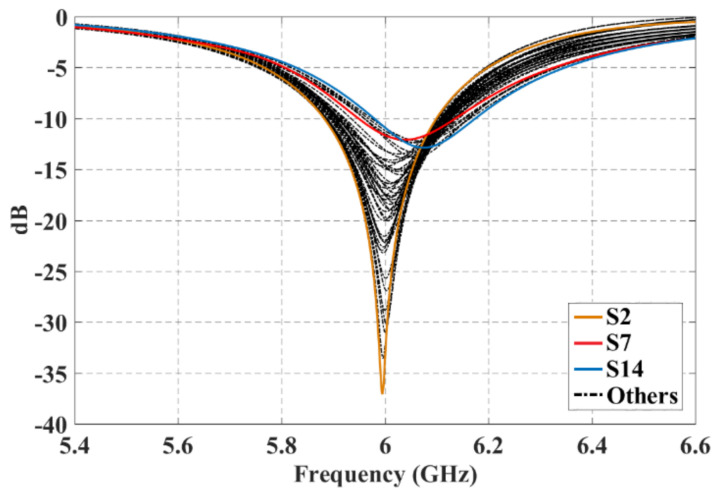
Active reflection coefficients for the 49 antenna elements of the 7 × 7 planar array of the configuration 1 at $\phi_{0}=90{}^{\circ}$ and $\theta_{0}=40{}^{\circ}$.

Every scanning direction was examined for all of these three configurations. All of the cases presented a good matching performance and the reflection coefficient was lower than −10 dB in the frequency of interest. The case of worst performance for active reflection coefficients was configuration 1 for the furthest scanning direction. Figure 10 shows the active reflection coefficients for the 49 antenna elements of configuration 1 when the beam is scanned to $\phi_{0}=90{}^{\circ}$ and $\theta_{0}=40{}^{\circ}$. As shown in this figure for the case of worst performance and the farthest direction the active reflection coefficient of 49 antenna elements is remained below −10 dB (for all scanning directions) at 6 GHz.

**Figure 11:**
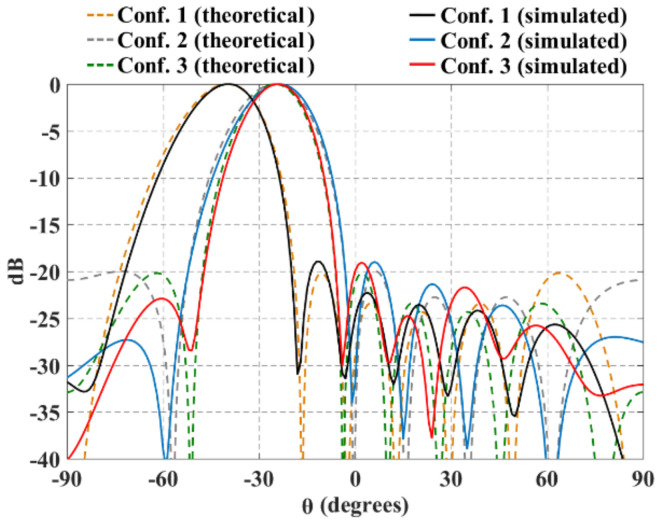
Radiation pattern in azimuth for the furthest scanning direction ($\phi_{0}=90{}^{\circ}$, $\theta_{0}=-40{}^{\circ}$) for configuration 1, ($\phi_{0}=90{}^{\circ}$, $\theta_{0}=-25{}^{\circ}$) for configuration 2 and ($\phi_{0}=90{}^{\circ}$, $\theta_{0}=-25{}^{\circ}$) for configuration 3.

**Figure 12:**
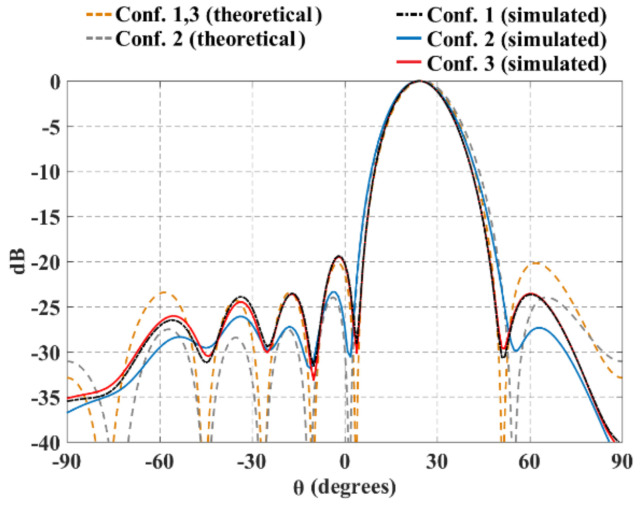
Radiation pattern in elevation for the furthest scanning direction of the three planar array configurations ($\phi_{0}=0{}^{\circ}$ and $\theta_{0}=25{}^{\circ}$).

Furthermore, Figure 11 and Figure 12 illustrate the radiation pattern obtained theoretically (without mutual coupling) and by using full-wave simulation (CST Microwave Studio) for the three configurations shown in Figure 1. Figure 11 shows the behavior of the radiation pattern in azimuth for the furthest scanning direction (from the natural response of the array), i.e., ($\phi_{0}=90{}^{\circ}$, $\theta_{0}=-40{}^{\circ}$) for configuration 1, ($\phi_{0}=90{}^{\circ}$, $\theta_{0}=-25{}^{\circ}$) for configuration 2 and ($\phi_{0}=90{}^{\circ}$, $\theta_{0}=-25{}^{\circ}$) for configuration 3. Furthermore, Figure 12 shows the behavior of the radiation pattern in the elevation plane for the furthest scanning direction from broadside ($\phi_{0}=0{}^{\circ}$ and $\theta_{0}=25{}^{\circ}$) of the three configurations. As shown in these figures, the full-wave simulation results agree theoretical results. The full-wave simulations obtained by electromagnetic solver present a $SLL_{PEAK}\;\approx -19$ dB for the configurations 1 and 3, and $SLL_{PEAK}\;\approx -23$ dB for configuration 2. Configuration 2 presents a lower *SLL* due to the lower value of *a* (in the equation 7).

The maximum performance deterioration in *SLL* is 1.1 dB for configuration 1 ($\phi_{0}=90{}^{\circ}$ y $\theta_{0}=-40{}^{\circ}$) and ≈1.0 dB for configurations 2 and 3. This is due to the mutual coupling among elements and the errors caused by the feeding network (amplitude and phase). As expected, the configuration 2 generates a higher beam-width due to have less antennas with respect to the configurations 1 and 3. As expected and shown in previous figures, the results illustrate a good performance for the three configurations of planar antenna arrays. The performance is remained during beam-scanning in the ranges previously mentioned.

**Figure 13:**
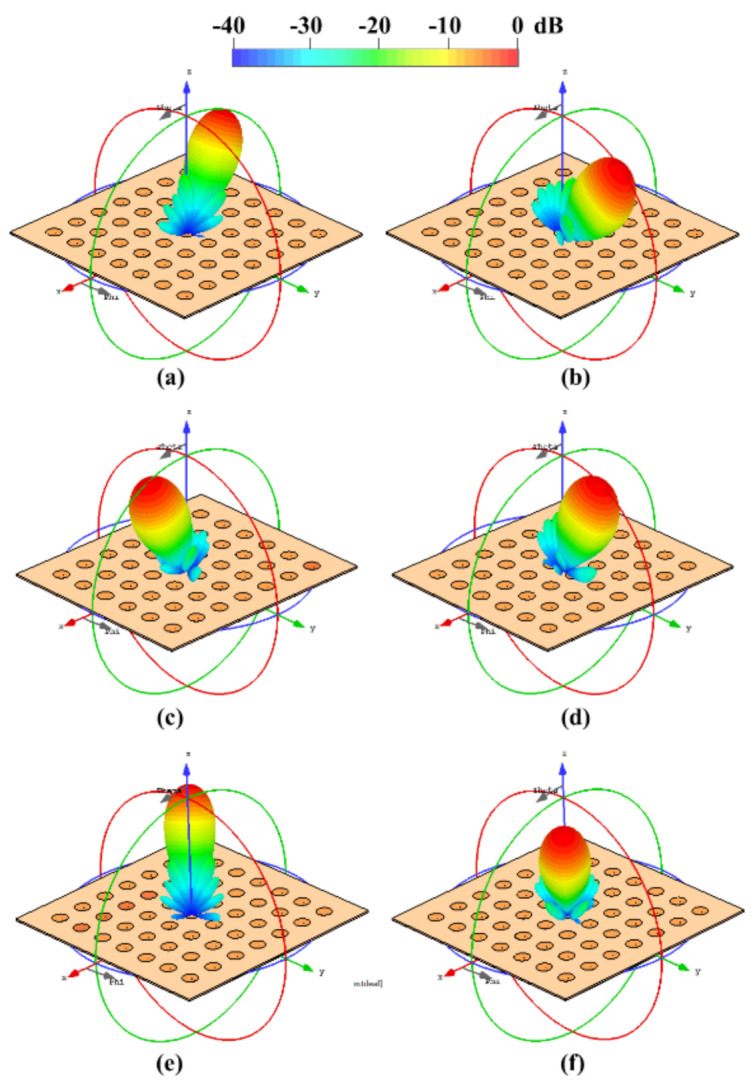
3D radiation pattern obtained by CST electromagnetic solver: configuration 1 at ($\phi_{0}=0{}^{\circ}$, $\theta_{0}=25{}^{\circ}$) (**a**), and ($\phi_{0\;}=90{}^{\circ}$, $\theta_{0}=40{}^{\circ}$) (**b**); configuration 2 at ($\phi_{0}=0{}^{\circ}$, $\theta_{0}=25{}^{\circ}$) (**c**), and $\phi_{0}=90{}^{\circ}$, $\theta_{0}=25{}^{\circ}$ (**d**); configuration 3 at ($\phi_{0}=225{}^{\circ}$, $\theta_{0}=25{}^{\circ}$) (**e**), and ($\phi_{0}=45{}^{\circ}$, $\theta_{0}=25{}^{\circ}$) (**f**).

Figure 13 shows the three planar array configurations and the 3D radiation pattern obtained by the CST electromagnetic solver. This figure illustrates the next cases: configuration 1 at $\phi_{0}=0{}^{\circ}$ and $\theta_{0}=25{}^{\circ}$ (Figure 13a), and $\phi_{0}=90{}^{\circ}$ and $\theta_{0}=40{}^{\circ}$ (Figure 13b); configuration 2 at $\phi_{0}=0{}^{\circ}$ and $\theta_{0}=25{}^{\circ}$ (Figure 13c), and $\phi_{0}=90{}^{\circ}$ and $\theta_{0}=25{}^{\circ}$ (Figure 13d); configuration 3 at $\phi_{0}=225{}^{\circ}$ and $\theta_{0}=25{}^{\circ}$ (Figure 13e), and $\phi_{0}=45{}^{\circ}$ and $\theta_{0}=25{}^{\circ}$ (Figure 13f). These results illustrate that the *SLL* performance is remained below −19 dB for all scanning directions analyzed.

**Figure 14:**
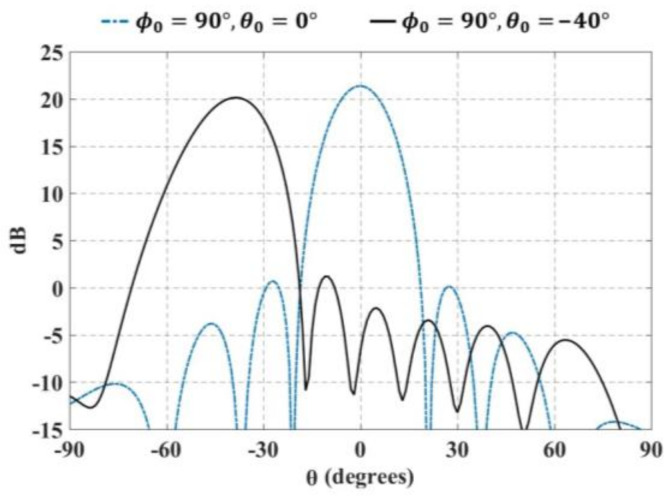
Gain loss during beam scanning for the radiation pattern.

The case of worst performance for gain of the 2-D array during beam scanning is obtained for the configuration 1 (i.e., a value of 1.3 dB is obtained as the gain loss for the configuration 1 as the worst case). Figure 14 illustrates the gain loss during beam scanning for the radiation pattern.

**Figure 15:**
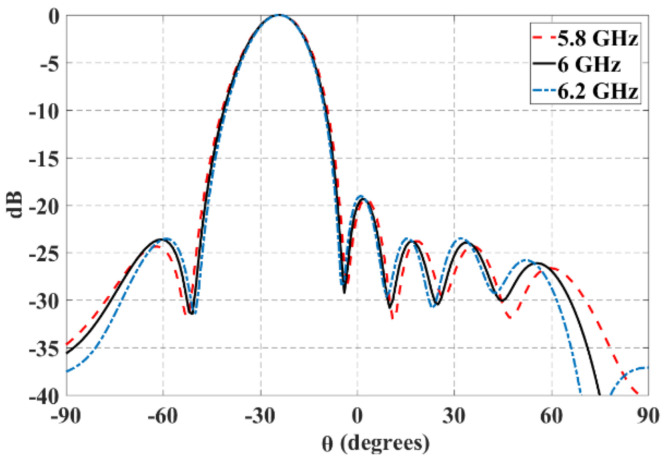
Normalized radiation pattern at 5.8 GHz, 6 GHz, and 6.2 GHz and for *θ*_0_ = −25°.

Figure 15 shows the normalized radiation pattern at 5.8 GHz, 6 GHz and 6.2 GHz and for *θ*_0_ = −25°. As shown in this figure, the radiation pattern changes very slightly with the changes in the frequency values.

Table 5 illustrates a comparative analysis of the three proposed configurations with respect to other existing techniques for 2-D phased arrays. This comparative analysis considers the reduction of PS devices, number of elements, elevation scanning range, azimuth scanning range, and peak side lobe level. The proposed design configurations present a reduction in PS devices of 45%, 64%, and 69% for configuration 1, configuration 2 and configuration 3, respectively. The proposed configurations, for this reduction capability of PS devices, provide a good design compromise in terms of the peak side-lobe level (−19 dB obtained by full-wave simulations considering mutual coupling among antenna elements) and scanning range with respect to other cases in the state-of-the-art.

## 4. Conclusions

This paper illustrated the application of CORPS for feeding 2-D phased arrays with a reduced number of phase shifter (PS) devices. The proposed design configurations for feeding the antenna elements in the planar array geometry provided several advantages with respect to others (in the state of art) in the scanning capability and the reduction of the number of PS devices of the array system. The full-wave simulation results for the proposed configurations in 2-D phased arrays provided a reduction in the number of PSs of until 69% for a scanning range of ±25° in elevation and until ±40° in azimuth. The application of the raised cosine amplitude distribution provided a good design value in terms of the peak side-lobe level of −19 dB considering mutual coupling among antenna elements.

Only the simulation results were presented without fabrication and measurement, but the originality of the proposed structure itself is recognized.

## Figures and Tables

**Table 1:** Distribution of raised cosine amplitude proposed for the 7 × 7 planar array considering a value of a = 0.19 and a ${\mathrm{SLL}}_{\mathrm{PEAK}}$ = −20 dB.

$I_{n,m}$	$I_{n,1}$	$I_{n,2}$	$I_{n,3}$	$I_{n,4}$	$I_{n,5}$	$I_{n,6}$	$I_{n,7}$
$I_{1,m}$	0.19	0.34	0.45	0.49	0.45	0.34	0.19
$I_{2,m}$	0.34	0.54	0.69	0.75	0.69	0.54	0.34
$I_{3,m}$	0.45	0.69	0.87	0.93	0.87	0.69	0.45
$I_{4,m}$	0.49	0.75	0.93	1.00	0.93	0.75	0.49
$I_{5,m}$	0.45	0.69	0.87	0.93	0.87	0.69	0.45
$I_{6,m}$	0.34	0.54	0.69	0.75	0.69	0.54	0.34
$I_{7,m}$	0.19	0.34	0.45	0.49	0.45	0.34	0.19

**Table 2:** Distribution of raised cosine amplitude proposed for the 7 × 6 planar array considering a value of a = 0.14 and a ${\mathrm{SLL}}_{\mathrm{PEAK}}$ = −20 dB.

$I_{n,m}$	$I_{n,1}$	$I_{n,2}$	$I_{n,3}$	$I_{n,4}$	$I_{n,5}$	$I_{n,6}$
$I_{1,m}$	0.14	0.28	0.37	0.37	0.28	0.14
$I_{2,m}$	0.32	0.54	0.67	0.67	0.54	0.32
$I_{3,m}$	0.48	0.75	0.91	0.91	0.75	0.48
$I_{4,m}$	0.54	0.83	1.00	1.00	0.83	0.54
$I_{5,m}$	0.48	0.75	0.91	0.91	0.75	0.48
$I_{6,m}$	0.32	0.54	0.67	0.67	0.54	0.32
$I_{7,m}$	0.14	0.28	0.37	0.37	0.28	0.14

**Table 3:** Transmission and reflection values for the block of 4 × 7 CORPS [10].

		Measured Value	Simulated Value
Bandwidth (−10 dB)		2.60 GHz	2.63 GHz
Min. frequency value (bandwidth)		4.76 GHz	4.55 GHz
Max. frequency value (bandwidth)		7.36 GHz	7.18 GHz
Max. reflection value at 6 GHz in all input ports		−19.62 dB	−23.1 dB
Transmission values at 6 GHz	S1,5	−8.48 dB	−8.21 dB
	S1,6	−12.07 dB	−11.77 dB
	S2,6	−11.98 dB	−11.72 dB
	S2,7	−13.12 dB	−11.73 dB
	S2,8	−14.94 dB	−14.93 dB
	S3,8	−15.40 dB	−15.79 dB
	S3,9	−12.72 dB	−11.90 dB
	S3,10	−10.66 dB	−11.66 dB
	S4,10	−11.08 dB	−11.77 dB
	S4,11	−8.68 dB	−8.20 dB

**Table 4:** Maximum values of the variable amplifiers and maximum values of phase error for each configuration.

Design Case	Maximum Value of Variable Amplification	Maximum Value of Phase Error
Configuration 1	7.98	9.4°
Configuration 2	8.89	9.6°
Configuration 3	11.28	10.7°

**Table 5:** Comparison of the three proposed configurations with respect to other existing techniques for 2-D phased arrays.

	Number of Elements	Number of PS Devices	Reduction of Phase Shifters (%)	Number of Variable Amplifiers	Number of Fixed Amplifiers	Elevation Scanning Range	Azimuth Scanning Range	Peak Side Lobe Level
Conventional phased array (raised cosine taper)	49	49	0%	0	49	± 42°	± 42°	−20 dB (AF)
This work (Configuration 1)	49	27	45%	21	28	± 25°	± 40°	−19 dB (Full-wave)
This work (Configuration 2)	42	15	64%	26	16	± 25°	± 25°	−19 dB (Full-wave)
This work (Configuration 3)	49	15	69%	33	16	± 25°	± 25°	−19 dB (Full-wave)
[5]	768	192	75%	0	192	± 10°	± 45°	−18 dB (Full-wave)
[1]	256	60	76%	0	60	± 15°	± 40°	−12 dB (AF)
[9]	9	4	55%	0	0	± 14°	± 12°	−11 dB (Meas.)
[17]	9	4	55%	0	0	± 14.5°	± 14.5°	−9.5 dB (Meas.)
